# Supplementary material for: Content-rich biological network constructed by mining PubMed abstracts
Source: BMC Bioinformatics. 2004 Oct 8;5:147. doi: 10.1186/1471-2105-5-147 (PMC528731; doi:10.1186/1471-2105-5-147)
Supplement: Additional File 5 — The original Chilibot query results of the term "long-term potentiation (LTP)" and 22 other terms, limiting the latest references analyzed to the years 1990, 1995, 2000, and 2004. [file 1471-2105-5-147-S5.bz2 › chilibotAdditionalFile5/ltp2000/html/ERK_ACTININ.html]

 


 **ERK** and **ACTININ** 
  
Found 3 abstracts in PubMed,  **3 abstracts were retrieved and analyzed**.  


---

 Search Google  |
 PDF files only 
|  EDU domain only 

---

**Interactive relationship** (e.g. stimulation, inhibition, etc)

- **ERK**  also bound, in a gel overlay assay, alpha  **actinin** , a protein with two tandem CH domains, as well as a 27 kDa thermolysin product of alpha  **actinin**  containing the CH domains of alpha  **actinin** .  Ref: 10548541 Biochem J, 1999

**Parallel relationship** (e.g. studied together, co-existance, homology, etc.)

- The CH domain of calponin could compete with intact calponin or alpha  **actinin**  for  **ERK**  binding.  Ref: 10548541 Biochem J, 1999
